# Tattoo Delivery of a Semliki Forest Virus-Based Vaccine Encoding Human Papillomavirus E6 and E7

**DOI:** 10.3390/vaccines3020221

**Published:** 2015-03-24

**Authors:** Stephanie van de Wall, Mateusz Walczak, Nienke van Rooij, Baukje-Nynke Hoogeboom, Tjarko Meijerhof, Hans W. Nijman, Toos Daemen

**Affiliations:** 1 Department of Medical Microbiology, University of Groningen, University Medical Center Groningen, HPC EB88, PO Box 30.001, 9700 RB Groningen, The Netherlands; E-Mails: m.n.s.van.de.wall@umcg.nl (S.W.); Mateusz.Walczak@rr-research.no (M.W.); n.van.rooij@umcg.nl (N.R.); b.n.hoogeboom@umcg.nl (B.-N.H.); t.meijerhof@umcg.nl (T.M.); 2 Department of Obstetrics & Gynecology, University of Groningen, University Medical Center Groningen, 9700 RB Groningen, The Netherlands; E-Mail: h.w.nijman@umcg.nl

**Keywords:** tattooing, viral vector, immunotherapy, cervical cancer

## Abstract

The skin is an attractive organ for immunization because of the presence of antigen-presenting cells. Intradermal delivery via tattooing has demonstrated superior vaccine immunogenicity of DNA vaccines in comparison to conventional delivery methods. In this study, we explored the efficacy of tattoo injection of a tumor vaccine based on recombinant Semliki Forest virus replicon particles (rSFV) targeting human papillomavirus (HPV). Tattoo injection of rSFV particles resulted in antigen expression in both the skin and draining lymph nodes. In comparison with intramuscular injection, the overall antigen expression determined at the site of administration and draining lymph nodes was 10-fold lower upon tattoo injection. Delivery of SFV particles encoding the E6 and E7 antigens of human papillomavirus type 16 (SFVeE6,7) via tattooing resulted in HPV-specific cytotoxic T cells and *in vivo* therapeutic antitumor response. Strikingly, despite the observed lower overall transgene expression, SFVeE6,7 delivered via tattoo injection resulted in higher or equal levels of immune responses as compared to intramuscular injection. The intrinsic immunogenic potential of tattooing provides a benefit for immunotherapy based on an alphavirus.

## 1. Introduction 

Vaccination in the form of viral vectors serves as a potent strategy for the induction of CD8^+^ T cell responses in the context of various diseases. In our group, a therapeutic immunization strategy based on an alphavirus, Semliki Forest virus (SFV), has been developed for targeting cervical cancer and premalignant cervical lesions induced by Human Papillomavirus (HPV). We have generated recombinant SFV replicon-based particles (rSFV) encoding a fusion protein of E6 and E7 from HPV type 16 (rSFVeE6,7). We demonstrated that intramuscular immunization of mice with rSFVeE6,7 particles results in strong HPV-specific cellular responses with eradication of established HPV-transformed tumors [1,2]. These strong responses are also induced in immune-tolerant mice and the efficacy of SFVeE6,7 immunization is not hampered by immunosuppressive regulatory T cells [3]. The intrinsic potency of alphavirus vectors has great promise for success in the clinic. However, clinical responses are often met with insufficient protection against cancer occurrence where the potency of the vector alone may not be sufficient for protection. Vaccine efficacy may be increased by alternative delivery methods. 

The skin is a complex, immunologically rich organ providing a first line of defense against a wide range of pathogens. Antigen-presenting cells (APCs), such as dendritic cells (DCs), are part of innate immunity that patrol the body from densely rich areas in the skin. The skin is divided into two main layers, the epidermis and the dermis [4]. The epidermis is the outermost layer and consists mainly of keratinocytes. This layer is rich in Langerhans cells, APCs that can efficiently process and present antigen to T cells [5,6,7]. The dermis lies underneath the epidermis, consisting mostly of collagen. It is made up of a very dense network of capillary blood and lymphatic vessels in which dermal dendritic cells, monocytes, polymorphonuclear lymphocytes, and mast cells circulate [8,9]. Given the diversity of immune cells in the skin that shape the adaptive immune response, this organ is an attractive site for immunization. 

Intradermal injection of vaccines based on DNA, protein, attenuated live bacteria and inactivated virus induces both humoral and cellular immune responses [10,11,12,13,14]. Intradermal (i.d.) delivery through conventional means can be achieved through syringe injection. However, standard i.d. delivery is focused on a single injection at one spot. Enhancing vaccine delivery at multiple sites is achieved by use of tattooing or microneedle arrays [4,15,16,17]. Tattooing is an attractive method delivery mainly explored within the context of DNA and peptide-based vaccines [18,19,20,21]. Conventional DNA vaccines elicit higher cellular immune responses in mice and non-human primates when delivered with a tattoo device compared to intramuscular or subcutaneous injection, circumventing the requirement of additional adjuventation [22,23,24]. Although DNA tattooing has been described to induce potent immune responses, little is known about the efficacy of tattooing with viral vectors. Up till now only one paper reported on an adenovirus-based vaccine administered by tattooing [25]. In the present study, we evaluated the efficacy of the SFVeE6,7 vaccine upon skin tattooing in the murine model system and compared it to that of responses induced by intramuscular injection. Next, therapeutic efficacy in tumor challenge experiments and memory T cell responses induced by SFVeE6,7 tattoo immunization was examined. To our knowledge this is the first study describing the efficacy of immune responses induced by an alphavirus-based vaccine administered via tattoo injection.

## 2. Materials and Methods 

### 2.1. Cell Lines

Baby hamster kidney cells (BHK-21) were obtained from the American Type Culture Collection (#CCL-10). C3 cells, 13-2 cells and TC-1 cells were a kind gift from Dr. Cornelis J. Melief and Dr. Rienk Offringa (Leiden University Medical Center, The Netherlands). The C3 cell expresses the complete HPV16 genome, while 13-2 cells express the HPV16 E7 CTL epitope, AA 49-57 (RAHYNIVTF). The TC-1 cell line was generated from C57Bl/6 primary lung epithelial cells with a retroviral vector expressing HPV16 E6E7. All cells were cultured as described before [26]. 

### 2.2. Mice

Specified pathogen-free female C57BL/6 mice were used at 8 to 12 weeks of age at the onset of the experiment. They were purchased from Harlan CPB (Zeist, The Netherlands) and kept according to institute guidelines. All animal experiments were approved by the local Animal Experimentation Ethical Committee.

### 2.3. Production, Purification and Titer Determination of SFV-EGFP, SFVLuc and SFVeE6,7 Particles

The production, purification and titer determination of SFV-EGFP, SFVLuc and SFVeE6,7 was performed as described previously [27]. In brief, SFV-EGFP, SFVLuc and SFVeE6,7 particles were produced by the co-electroporation of BHK-21 cells with an RNA encoding the SFV replicase and the transgene (enhanced green fluorescent protein, firefly luciferase or a E6E7 fusion protein) and a helper RNA encoding the structural proteins of SFV. The rSFV replicon particles produced by the transfected BHK-21 cells were purified on a discontinuous sucrose density gradient. Before use, rSFV particles were activated with α-chymotrypsin (Sigma Chemical Co., St. Louis, MO, USA) at the final concentration of 0.5 mg/mL to cleave the mutated spike proteins. Subsequently, α-chymotrypsin was inactivated by the addition of aprotinin (Sigma Chemical Co.) at the final concentration of 1 mg/mL. rSFV particles were titrated on BHK-21 cells using a polyclonal rabbit antireplicase (nsP3) antibody (a kind gift from T. Ahola (Biocentre Viiki, Helsinki, Finland)) [27]. 

### 2.4. SFV-EGFP, SFVLuc and SFVeE6,7 Administration

To determine the quantity of antigen expression at the site of injection, mice were tattooed or intramuscularly injected with 10^7^ infectious units (i.u.) of SFVLuc or SFV-EGFP. The tattoo injection was adapted from the method by Bins *et al.* for DNA administration [22]. Briefly, one day before immunization, the hair on the hind legs was removed by depilating cream (Veet^®^, Reckitt Benckiser, Hull, UK). After depilation, the skin was washed thoroughly with warm water to avoid irritation from the depilating cream. The following day, rSFV was applied on the skin (10 μL per leg) and the skin was tattooed for 30 s using a Symphony tattoo device (a kind gift from Mt. Derm GMBH, Berlin, Germany) with 9-needle magnum cartridge with a needle depth of 1 mm and oscillating at a frequency of 100 Hz. In the immunization experiments, the mice were immunized by tattoo and/or intramuscular injection with 5 × 10^6^ i.u. of SFVeE6,7, followed by one booster immunization two weeks later. 

### 2.5. Imaging of Luciferase Expression

Luciferase expression was measured *in vivo* using bioluminescence imaging with an ultra-sensitive charge-coupled device (CCD) camera within the *In Vivo* Imaging System IVIS 100^®^ (Xenogen, Alameda, CA, USA), at 6 h and 30 h after SFVLuc administration. Mice were anesthetized with isoflurane and 10 min before imaging, mice were injected intraperitonally with D-luciferin (Xenogen). Mice were placed into the chamber of the CCD camera and images of the luciferase distribution in the body were generated with an integration time of 30 s. Later, pseudocolor images representing light intensity (blue—least intense; red—most intense) were created using The Living Image^®^ (Xenogen) and Igor Pro^®^ (WaveMetrics, Lake Oswego, OR, USA).

### 2.6. Quantification of Luciferase Expression

Hind limb muscles, the tattooed skin region and inguinal lymph nodes were isolated to quantify luciferase expression *ex vivo* 6 h and 30 h after rSFV injection. Immediately after excision, the organs were frozen in liquid N_2_ and kept at −80 °C. The organs were crunched to powder in a mortar on dry ice. The material was lysed in lysis buffer (Promega, Leiden, The Netherlands) by three freeze-thaw cycles with vigorous vortexing in between. Cell debris was removed by centrifugation. Immediately before measurement, lysates of the samples were mixed with luciferase substrate solution (Promega). The luciferase signal was determined in a luminometer (Synergy HT, BioTek, Winooski, VT, USA). Background intensity was subtracted from the signal of measured samples.

### 2.7. *Ex Vivo* Detection of EGFP Expression

Skin and muscle from the site of vaccination were isolated and fixed in 4% paraformaldehyde and 10% sucrose in PBS at 4 °C for 30 min to overnight. Tissues were further embedded in Tissue-Tec^®^ O.C.T (Sakura Finetek, Torrance, CA, USA) above liquid nitrogen. Cryosections of 4 μm were cut from the frozen tissue blocks and mounted on poly-l-lysine-coated slides. Slides were dried and stored at −20 °C until use. 

Slides were brought to RT and placed in PBS for 5 min to remove the OCT and stained with rabbit anti-GFP (Abcam, Cambridge, UK). Anti-rabbit FITC (Abcam) was used as a secondary antibody. Slides were mounted with Prolong Gold antifade containing DAPI (Invitrogen, Carlsbad, CA, USA). Staining was evaluated by fluorescence microscopy.

### 2.8. MHC Class I Tetramer Staining 

To determine the number of CD8^+^ T cells that are specific for the HPV16 E7_49__–__57_ peptide RAHYNIVTF, 10^6^ freshly isolated splenocytes and inguinal lymph node cells were washed with FACS buffer (PBS containing 0.5% bovine serum albumin) and stained with PE-conjugated H2-Db RAHYNIVTF tetramers. PE-labeled tetramers were obtained from LUMC, Leiden, The Netherlands. Subsequently, the cells were stained with FITC-conjugated anti-CD8 (ProImmune, Oxford, UK). Cells were washed twice and analyzed by flow cytometry using LSR-II (BD Biosciences, Erembodegem, Belgium). Dead cells were excluded by LIVE/DEAD^®^ Fixable Dead Cell Stain Kit (Invitrogen Ltd., Paisley, United Kingdom).

### 2.9. Regular CTL and Micro-CTL Assay

For the regular CTL assay, spleen cells were isolated 10 days after the booster immunization and co-cultured with irradiated (100 gray) TC-1 cells in a ratio of 25:1 (20 × 10^6^ spleen cells and 0.8 × 10^6^ irradiated TC-1 cells). Cells were co-cultured in IMDM-complete medium (Iscove’s modified DMEM with Glutamax (Gibco, New York, NY, USA) 10% FCS (Bodinco B.V., Alkmaar, The Netherlands), 100 U/mL penicillin (Invitrogen, Paisley, UK), 100 μg/mL streptomycin (Invitrogen), 50 μM β-mercaptoethanol (Sigma, St. Louis, MO, USA)) in 25 cm^2^ culture flasks, placed upright. Recombinant IL-2 (Peprotech, New York, NY, USA) was added to the cells on day 5 of culture. After a 7-day *in vitro* restimulation culture, the cells were harvested and CTL activity was determined in a standard 4 h ^51^Cr release assay. In the micro-CTL assay, spleen or lymph node cells were co-cultured with irradiated (100 gray) TC-1 cells in a ratio 25:1 (0.5 × 10^6^ spleen or inguinal lymph node cells and 0.02 × 10^6^ irradiated TC-1 cells/well) in IMDM-complete medium in 96-wells plates. Recombinant IL-2 (Peprotech) was added to the cells on day 5 of culture. After 7 days *in vitro* restimulation, cells in the micro-CTL assay were not harvested, but ^51^Cr-labeled (MP Biomedicals, Inc., Irvine, CA, USA) target cells (C3 cells) were directly added to the cultures. Two days before the ^51^Cr release assay, recombinant IFN-γ (Peprotech) was added to the C3 cell cultures. The mean percentage of specific ^51^Cr-release was calculated according to the formula: % specific release = ((experimental release − spontaneous release)/(maximal release − spontaneous release)) cpm × 100. The spontaneous ^51^Cr-release was always <15%. The standard errors of the means of the triplicate determinations were <10% of the mean.

### 2.10. IFN-γ Elispot Assay

ELISA plates (Greiner, Alphen, The Netherlands) were coated overnight with purified anti-mouse IFN-γ (BD Pharmingen, San Jose, CA, USA) in coating buffer (0.1M Na_2_HPO_4_, pH 9.0) at 37 °C. After washing with PBST (PBS containing 0.02% Tween-20), the plates were incubated with blocking buffer (PBS containing 4% RIA grade bovine serum albumin (Sigma, St. Louis, MO, USA)) for at least 1 h at 37 °C. Freshly isolated spleen or inguinal lymph node cells were serially diluted in IMDM-complete medium containing 5% FCS and plated into the wells. The splenocytes or inguinal lymph node cells were incubated overnight with serial dilutions of 100 Gy irradiated stimulator 13-2 cells at 37 °C. Next day, the cells were lysed by addition of ice-cold water for 10 min. After washing with PBST, plates were incubated with biotinylated anti-mouse IFN-γ mAb (BD Pharmingen, San Jose, CA, USA) for 1 h at 37 °C. Subsequently, plates were washed and incubated with streptavidin-alikaline phosphatase (BD Pharmingen, San Jose, CA, USA) for 1 h at 37 °C. Spots were developed by adding substrate (1 mg/mL 5-Bromo-4-chloro-3-indolyl phosphate in 125 mM MgCl_2_, 6 mg/mL agarose, 9.2 mg/mL 2-amino-2-methyl-1-propanol and 0.08 μL/mL Triton X-405 (all from Sigma)) and incubated for 45 min at 37 °C. The spots were counted in triplicate with the A.EL.VIS spot analyzer (Sanquin, Amsterdam, The Netherlands). Background from unstimulated cells was subtracted.

### 2.11. Tumor Treatment Experiments

Mice were inoculated subcutaneously in the neck with 2 × 10^4^ TC-1 cells suspended in 0.2 mL Hank’s Balanced Salt Solution (Invitrogen, Paisley, UK). Seven days later the mice were tattoo or intramuscularly immunized with 10^5^ or 5 × 10^6^ i.u. of SFVeE6,7 and boosted twice with a one-week interval (day 14 and 21). Control mice were injected intramuscularly with PBS. The same skilled technician performed the tumor measurements. When the tumor grew through the skin or if the tumor volume exceeded 1000 mm^3^, the mice were killed.

### 2.12. Statistical Analysis

Data are presented as the mean ± SD. Data were analyzed using Student *t*-Test. The Cox proportional hazards test was used for statistical analysis of tumor treatment responses. Statistical significance was defined as *p* < 0.05.

## 3. Results 

### 3.1. Transgene Expression upon Tattoo Injection of rSFV Replicon Particles

**Figure 1:**
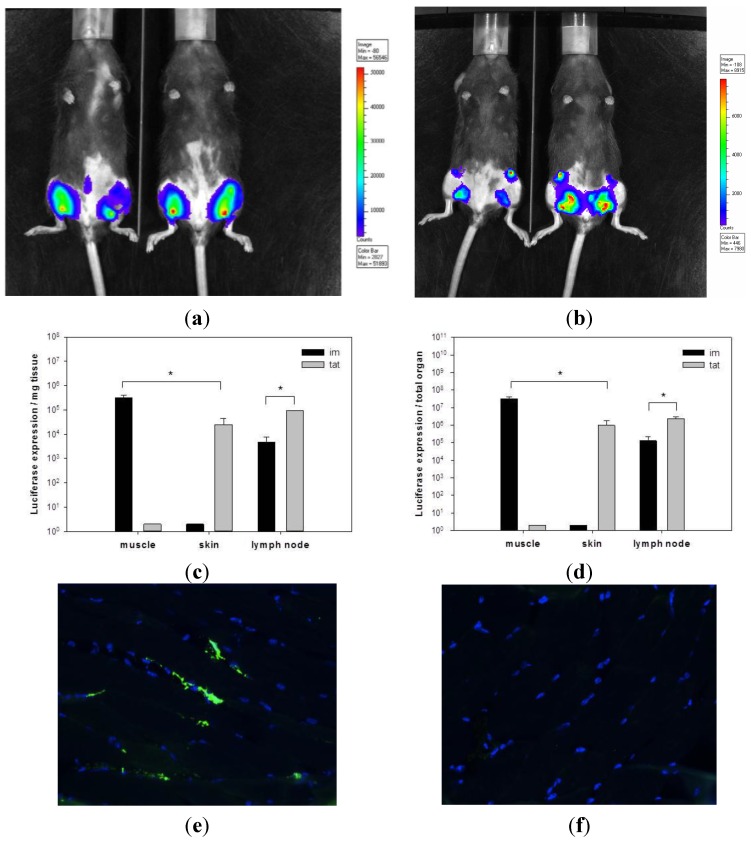
Tattooing of rSFV replicon particles results in high transgene expression. Luciferase expression by SFVLuc was determined *in vivo* with bioluminescence imaging using a CCD camera. Mice were tattooed (**a**) or injected intramuscularly (**b**) with 10^7^ i.u. of SFVLuc. After 6 h, images with an integration time 30 s were taken. Blue colors represent the least and red colors represent the most intense light intensity. Two representative mice for each group are shown. Immediately after *in vivo* measurements, animals were sacrificed and muscles from the intramuscularly treated group or skin from the tattooed group and draining inguinal lymph nodes were isolated for *ex vivo* luciferase expression. Luciferase expression is shown as expression per mg organ (**c**) or per total organ (**d**) (*n* = 4). Data of a representative experiment out of two are shown. * *p* < 0.05. Mice were intramuscularly injected with 10^8^ i.u. of SFV-EGFP (**e**) or PBS (**f**). After 24 h, skin and muscle from the site of administration were excised and analyzed by immunofluorescence staining. For immunofluorescent staining, muscle tissue expressing EGFP were analyzed using anti-GFP antibody (green). Cell nuclei were detected using DAPI (blue) (magnification 40×). A representative image for each group is shown.

To investigate the level of antigen expression upon rSFV administration, rSFV particles encoding firefly luciferase were used (SFVLuc). Mice were tattooed or intramuscularly injected with 10^7^ i.u. of SFVLuc. Six hours after rSFV administration, *in vivo* luciferase expression was visualized using bioluminescence imaging. Interestingly, upon rSFV tattooing, luciferase was not only expressed in the tattooed skin but also in the draining inguinal lymph nodes (Figure 1a). In comparison, SFVLuc injected intramuscularly resulted mainly in luciferase expression in the injected muscle (Figure 1b).

Following the *in vivo* bioluminescence imaging the mice were sacrificed and muscle, skin and draining inguinal lymph nodes were isolated to *ex vivo* quantify for luciferase expression. Confirming the bioluminescence imaging, skin tattooing with SFVLuc particles resulted in a relatively high level of luciferase activity in the draining inguinal lymph nodes. SFVLuc replicon particles administered via tattooing resulted in approx. 20-fold (per mg tissue and total organ) higher luciferase expression in the draining inguinal lymph nodes compared to intramuscular injection (Figure 1c,d). However, at the administration site (muscle or skin), the luciferase level was 20-fold higher after SFVLuc intramuscular injection than after tattoo injection. The overall antigen expression (the sum of luciferase expression at the administration site and in the draining inguinal lymph nodes) was approx. 10-fold lower after SFVLuc tattoo injection than after intramuscular injection.

The antigen expression upon tattoo or intramuscular injection of rSFV particles was additionally represented at a cellular level with detection of GFP. GFP expression was assessed 24 h after SFV-EGFP injection in skin or muscle. Unfortunately, we were not able to detect GFP in skin cryosections. In muscle cryosections, GFP was expressed in approximately 10% of myocytes (Figure 1e). The control is provided in Figure 1f.

These data indicate that transgene expression upon tattooing results in lower expression levels at the site of injection compared to intramuscular injection. This is in contrast to higher levels of transgene activity in the draining lymph node with tattooing. Despite this, the overall level of luciferase activity is lower with tattooing as compared to intramuscular injection of rSFV. 

### 3.2. Induction of Cytotoxic T Lymphocytes upon Tattooing and Intramuscular Immunizations with SFVeE6,7

**Figure 2:**
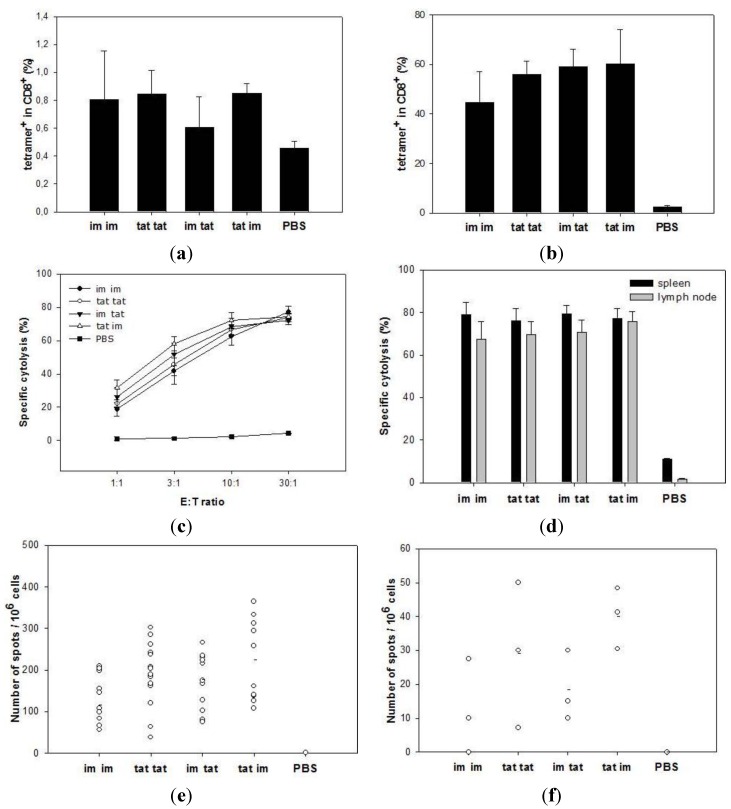
SFVeE6,7 tattoo injections induce strong T cell responses. Mice were tattooed or intramuscularly immunized with 5 × 10^6^ i.u. of SFVeE6,7 and boosted 14 days later using the same or alternative delivery route. Ten days after the booster immunization animals were sacrificed. Freshly isolated (**a**) and 7-day *in vitro* restimulated spleen cells (**b**) were stained with MHC class I tetramers and anti-CD8 antibodies and analyzed by flow cytometry. The percentages of tetramer-positive cells within the CD8 T cells are shown. Activity of restimulated spleen and inguinal lymph node cells were analyzed in regular (**c**); only spleen cells) and micro CTL assay (**d**; both spleen and lymph node cells). The frequencies of IFNγ-producing cells, both in the spleens and inguinal lymph nodes, were determined using Elispot assay. Results are expressed as number of IFNγ-producing cells per 10^6^ splenocytes (**e**) or per 10^6^ lymph node cells (**f**). Each dot represents an individual mouse; **a**–**d**—data from a representative experiment out of four (three mice/group); **e**—pooled data from four experiments; **f**—data from one experiment. E:T ratio—effector:target ratio. * *p* < 0.05 as compared with the im group.

Mice were immunized by tattooing or intramuscular injection with 5 × 10^6^ i.u. of SFVeE6,7 and boosted 14 days later using the same or the alternative route of administration. Animals were sacrificed ten days after the booster immunization. The frequency of HPV-specific CTLs was determined by direct staining of freshly isolated spleen cells with MHC class I tetramers and anti-CD8 antibodies. SFVeE6,7 tattoo prime and boost resulted in induction of HPV-specific CD8 T cells (approx. 0.8% RAHYNIVTF-specific cells in CD8 T cells; Figure 2a). Other immunization protocols resulted in comparable frequencies of antigen specific CD8 T cells. Tetramer staining after 7-day *in vitro* restimulation showed that CTLs induced *in vivo* with SFVeE6,7 tattooing readily proliferated *in vitro*, resulted in 50%–80% RAHYNIVTF-specific CD8 T cells (Figure 2b). These results were reflected in high CTL lytic activity of these cells as measured in a bulk ^51^Cr-release assay (Figure 2c). All immunization protocols used resulted in similar high levels of specific cytolysis (approx. 75% at an effector:target ratio of 30:1).

Cells from the inguinal lymph nodes were also isolated and *in vitro* restimulated to investigate their CTL activity. Here, a micro-CTL assay was used as the numbers of cells isolated from the lymph nodes were too small to perform a regular CTL assay. Cells isolated from SFVeE6,7 tattooed mice were characterized by high lytic activity (Figure 2d). CTL activity of *in vitro* restimulated lymph nodes cells was as high as CTL activity of *in vitro* restimulated splenocytes. No differences were observed between the different prime-boost strategies.

Spleen and draining inguinal lymph node cells were restimulated for 24 h before determining the number of HPV-specific IFNγ-producing cells in an ELISPOT assay. Interestingly, rSFV tattoo prime-boost immunization resulted in higher frequency of IFNγ-secreting cells in spleen compared to rSFV intramuscular prime-boost (Figure 2e; *p* < 0.05). Mice primed by tattooing and boosted intramuscularly were characterized by the highest frequency of IFNγ-specific cells (approx. 224 spots/10^6^ cells). On average, there were 8–10 fold less IFNγ-specific cells detected in the draining inguinal lymph nodes compared to the spleen (Figure 2f), but again, mice primed with tattoo injection and boosted intramuscularly were characterized by the highest frequency of IFNγ-specific cells in the draining inguinal lymph nodes (approx. 40 spots/10^6^ cells; *p* < 0.05 as compared to intramuscular prime-boost group). These results demonstrate that SFVeE6,7 administered via tattoo injection induces CTL responses which are superior to immune responses induced by intramuscular injection using the frequency of IFNγ-producing T cells as read-out.

### 3.3. Anti-Tumor Therapeutic Efficacy of SFVeE6,7 Delivered via Tattooing

**Figure 3:**
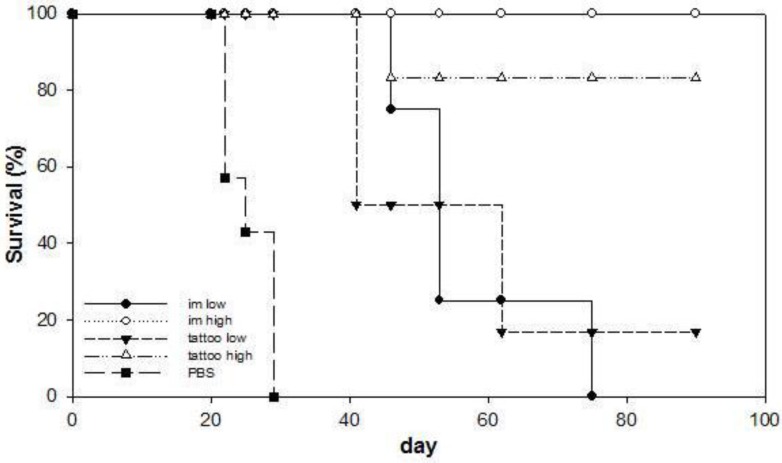
SFVeE6,7 tattoo immunizations result in high therapeutic efficacy. Mice were inoculated subcutaneously with 2 × 10^4^ TC-1 cells and 7 days later animals were tattoo- or intramuscularly immunized with 10^5^ (low dose) or 5 × 10^6^ (high dose) i.u. of SFVeE6,7 or injected with PBS. The mice were boosted twice with a one-week interval (day 14 and 21), using the same route of immunization. Tumor development was monitored twice weekly. When the tumor reached a volume of 1000 mm^3^, the mice were sacrificed. The effect of the immunization is depicted as the percentage of surviving mice. *n* = 7. * *p* < 0.05 as compared with PBS control group.

We analyzed anti-tumor therapeutic efficacy of SFVeE6,7 delivered via tattoo immunizations. Seven days after tumor inoculation mice were primed with 10^5^ or 5 × 10^6^ i.u. of SFVeE6,7 administered by tattooing or intramuscular injection. Mice were boosted twice, with a one-week interval using the same delivery route (day 14 and 21). Control mice developed tumors and had to be killed within 29 days after tumor inoculation (Figure 3). All SFVeE6,7 immunization protocols resulted in delayed tumor growth when compared to the control treatment (*p* < 0.05). In the groups immunized with 5 × 10^6^ i.u. of SFVeE6,7 via tattooing or intramuscular injections, almost all mice were tumor-free on day 90 after tumor inoculation. SFVeE6,7 immunization with a dose of 10^5^ i.u. resulted, as expected, in a lower therapeutic effect compared to 5 × 10^6^ i.u. of SFVeE6,7 particles with both immunization routes. These data indicate that immunization with SFVeE6,7 delivered via tattoo injection results in the induction of a potent therapeutic antitumor response.

### 3.4. Memory T Cells Induced by SFVeE6,7 Tattoo Immunizations

**Figure 4:**
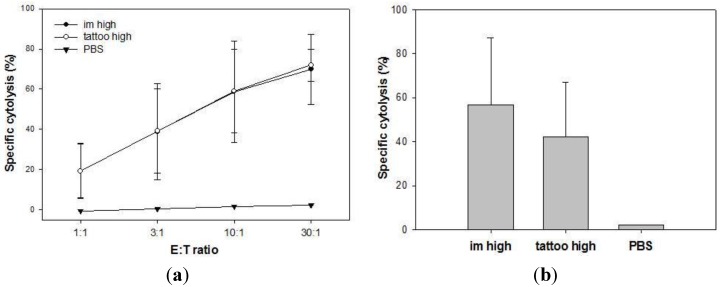
SFVeE6,7 tattooing induces long-lasting memory T cells. Mice that survived tumor challenge as depicted in Figure 3 were sacrificed on day 90 after tumor inoculation. Isolated spleen and inguinal lymph node cells were *in vitro* restimulated for seven days. Activity of restimulated spleen (**a**) and lymph node (**b**) cells were analyzed in regular and micro CTL assay, respectively. E:T ratio—effector:target ratio.

We further investigated the potency of SFVeE6,7 tattooing in inducing long-lasting T cell responses. Mice that survived the tumor challenge (depicted in Figure 3) were sacrificed on day 90 after tumor inoculation. Isolated spleen and draining inguinal lymph node cells were *in vitro* restimulated for seven days. Activity of restimulated spleen and lymph node cells was tested in regular and micro-CTL assays, respectively. Splenocytes isolated from each surviving mouse were able to efficiently lyse target cells after *in vitro* restimulation (Figure 4a). No differences were observed in lytic activity of spleen cells obtained from tattooed or intramuscularly injected mice. Similar results were obtained with cells isolated from draining inguinal lymph nodes analyzed with the micro-CTL assay (Figure 4b). These results show that SFVeE6,7 delivered via tattoo injection results in the induction of long-lasting T cells *in vivo*.

## 4. Discussion

In this study, a head-to-head comparison of tattoo and intramuscular administration with rSFV replicon particles was performed. The feasibility of tattoo administration and the immune response was determined with prime-boost immunizations. Interestingly, tattooing of rSFV particles induced higher transgene expression in the draining lymph nodes as compared to rSFV intramuscular injection. However, the overall transgene expression (the sum of transgene expression at the site of injection and in the draining lymph nodes) was 10-fold lower after rSFV tattooing compared to intramuscular injection. Despite this, SFVeE6,7 tattooing resulted in higher or equal HPV-specific IFNγ-producing cells, both in spleens and draining lymph nodes. These potent immune responses could not be further improved by combining different prime-boost immunization routes. Moreover, SFVeE6,7 delivered via tattooing was able to induce strong therapeutic antitumor effect *in vivo* and resulted in the generation of memory T cells in immunized mice.

Tattooing with rSFV replicon particles induced approx. 20-fold higher transgene expression levels in the draining lymph nodes compared to intramuscular injection using luciferase as a read-out. On the other hand, antigen expression levels in the skin upon rSFV tattooing were approx. 33-fold lower than the expression levels in the muscle after SFV intramuscular injection. Recombinant SFV particles are applied onto the skin in a very small volume and tattooed. Although the tattoo needle reservoir is pre-loaded with the vaccine, part of the applied material may be sucked into the needle reservoir. Thus, this is still a relatively uncontrolled procedure. To this effect, we expect that not all rSFV particles applied onto the skin will penetrate into the epidermis and/or dermis during the tattoo procedure. As described above, the overall antigen expression is approx. 10-fold higher after rSFV intramuscular injection compared to tattoo injection. Assuming an equal efficacy of rSFV infection in skin and muscle and assuming that 100% of the rSFV particles administered intramuscularly indeed transfect cells, this would mean that approximately 10% of the rSFV particles applied for tattooing actually enter the skin and transfect cells. This falls in line with the wide range of expression level observed by van den Berg *et al* [28]*.* The authors showed that DNA tattooing resulted in 10 to 100 lower antigen expression levels at the site of vaccination in comparison to intramuscular DNA injection. The expression levels upon tattooing of an adenovirus vector vaccine were also compared with simple intradermal injection [25]. Neither expression levels nor immune responses were significantly different between the two methods [25]. Simple intradermal injection also presents with inconsistency regarding expression in the dermis, limiting its use for clinical practice [29]. We therefore compared tattooing with standard intramuscular injection. 

Tattooing of SFVeE6,7 induced strong HPV-specific CTL responses in the spleen and lymph nodes as well as a potent antitumor therapeutic effect *in vivo*. CTL analysis of spleen and draining inguinal lymph nodes isolated from mice which survived TC-1 tumor challenge indicated that SFVeE6,7 administered via tattooing stimulated memory T cells formation. We were able to detect specific CD8 T cells in spleens and draining inguinal lymph nodes approx. 70 days after the last SFVeE6,7 tattoo immunization. For some vaccines it has been shown that they are especially potent in inducing immune responses when administered via different prime-boost routes. Here we demonstrate that mice primed with SFVeE6,7 tattooing and boosted intramuscularly (and *vice versa*) were characterized by potent CTL responses. Nevertheless, these responses were not higher than the ones induced by prime-boost protocols using the same delivery route.

Despite the differences in expression levels, there was no difference between the anti-tumor efficacy of tattoo and intramuscular injection of rSFV. This observation is reflective of a higher intrinsic immune potentiating capacity of intradermal delivery. This is likely due to the rich abundance of APCs in the epidermis and dermis such as Langerhans cells (LC) or dermal dendritic cells, respectively, which are known to be potent inducers of T-cell immunity. We were not able to demonstrate the preferred cell type at the skin and draining lymph node for SFV infection upon tattooing. Previously, we as well as others have demonstrated limited capacity of SFV to infect DCs *in vitro* [30,31,32]. Plausible candidates in the skin include fibroblasts or keratinocytes. Based on the morphology of the cells in the muscle infected with rSFV, these are the higher metabolically active myocytes. Regardless of the cell type infected, the cells eventually undergo apoptosis. The apoptotic bodies, which are loaded with antigen encoded by rSFV are taken up by antigen-presenting cells (APCs) and processed for antigen cross presentation [30,31]. We speculate that cross-presentation is most likely executed by lymph node resident CD8a^+^ DCs as previous studies have shown in the context of cross-presentation of antigen from apoptotic SFV-infected fibroblasts [30,33]. CD8^+^ DCs are also known to prime CTL immunity to viruses irrespective of the route of immunization as they are only DCs with specialized intracellular machinery to cross-present internalized antigens on MHC class I molecules [34,35,36,37]. These cells display approx. 10-fold higher cross-presentation efficiency than Langerhans cells [38]. 

The contribution of skin-derived DCs to the early expression of luciferase by rSFV in the lymph node is unlikely based on studies demonstrating that LC and dermal DCs take longer than 12 h to reach draining LN. It is more likely that as early as 6 h after tattooing, virus reaches the lymph nodes as free particles. Studies have shown that particles within the range of 20–200 nm directly reach the lymph nodes when either administered subcutaneously or intramuscularly [39]. This has also been demonstrated for skin delivery. For instance, Cubas *et al.* have shown that simian-human immunodeficiency (SHIV) virus-like particles, administered intradermally, primarily enter lymphatic vessels and reach the draining lymph nodes as intact particles [40]. As SHIV particles are approx. 90 nm in size and rSFV particles 65 nm, migration as intact particles may very well be the case for rSFV.

However, this does not discount the contribution that skin-derived DCs may have on immunogenicity (*i.e.*, independent of lymph node resident CD8^+^ DCs). At 24 h, Johnson *et al.* observed an increase in Langerhans cell density upon intradermal injection of wild-type SFV [41]. Despite the limited capacity of SFV to infect DCs *in vitro*, strategies have focused on targeting dendritic cells with the use of lentiviruses enveloped with glycoproteins derived from SFV by attaching to C-type lectins [42]. Langerhans cells may also shuttle virus particles by attachment to DC-SIGN, as observed in the case of HIV infection [43]. 

The initial immunogenic effect provided by tattooing is evidenced by the intradermal procedure itself, which in turn may activate resident DCs. Despite the slow migrating nature of Langerhans cells, migration may be an effect of cytokine production by keratinocytes having undergone mechanical stress by the thousands of epidermal punctures with tattooing [44]. The tattooing procedure itself results in a systemic interleukin-6 (IL-6) response that reaches higher levels than after an intraperitoneal injection with LPS [45]. IL-6, a pro-inflammatory cytokine, may be released by damaged keratinocytes [46,47]. The resultant acute inflammation can develop as early as 12 h with epidermal necrosis and hyperplasia in the lymph nodes. We have also observed an increase in lymph node size as early as 6 hours upon tattooing (data not shown). Even as early as 3 h, the acute inflammatory response initiated by IL-1β is elevated in the skin and claimed to be transported to the draining lymph node [48]. This cytokine has also been implicated in the migration of Langerhans cells in the context of other viruses [49].

In the last few years, appropriate adjuvants in combination with i.d. vaccines were extensively explored [50,51]. Adjuvants such as granulocyte-macrophage colony-stimulating factor (GM-CSF) and toll-like receptor 7 (TLR7) agonist imiquimod combined with tattooing resulted in no improvement in vaccine efficacy in preclinical studies [23,25]. However, it is difficult to extrapolate the effect of adjuvants, e.g., on induction of cross-presentation, from mouse to human due to the plasticity in the phenotype of DCs of both species. For instance, imiquimod applied topically (Aldara^®^) rather than intradermally enhances CD8^+^ T cell responses through human dermal DCs. This is presumably through an increase in cross-presentation as determined in a human explant model [52]. Human epidermal Langerhans cells have shown to efficiently cross-present CD8^+^ epitopes [53]. This was dependent on CD70 expression with blockade of CD70 signaling inhibiting priming of T cells. The influence of imiquimod is contradicted with Flacher *et al.* demonstrating that mice treated with topically with the adjuvant resulted in cross-tolerance with failure to prime CD8^+^ T cells. This was concomitant with a lack of CD70 upregulation in Langerhans cells [54]. In any case, these data suggest that targeting CD70 (e.g., by use of a CD27 agonist) may serve as a potential strategy in combination with our vaccine delivered intradermally for enhancing T cell priming and memory formation. 

## 5. Conclusions 

In conclusion, to our knowledge, we are the first to demonstrate that tattooing is an effective administration route for an alphavirus vector-based vaccine. Tattoo injection of SFV replicon particles results in a lower overall antigen expression compared to intramuscular injection. Yet, despite this lower overall antigen expression, SFVeE6,7 delivered via tattoo injection resulted in higher or equal levels of antitumor activity as observed after intramuscular injection. This result can be explained by the efficient intrinsic immune potentiating capacity of tattoo delivery. All in all, tattoo injection is an effective administration method for an alphavirus-based vector immunotherapy resulting in the induction of potent immune responses.

## References

[1] Riezebos-Brilman A., Walczak M., Regts J., Rots M.G., Kamps G., Dontje B., Haisma H.Y., Wilschut J., Daemen T. (2007). A comparative study on the immunotherapeutic efficacy of recombinant Semliki Forest virus and adenovirus vector systems in a murine model for cervical cancer. Gene Ther..

[2] Walczak M., de Mare A., Riezebos-Brilman A., Regts J., Hoogeboom B.N., Visser J.T., Fiedler M., Jansen-Durr P., van der Zee A.G., Nijman H.W. (2011). Heterologous prime-boost immunizations with a virosomal and an alphavirus replicon vaccine. Mol. Pharm..

[3] Walczak M., Regts J., van Oosterhout A.J., Boon L., Wilschut J., Nijman H.W., Daemen T. (2011). Role of regulatory T-cells in immunization strategies involving a recombinant alphavirus vector system. Antivir. Ther..

[4] Fehres C.M., Garcia-Vallejo J.J., Unger W.W., van Kooyk Y. (2013). Skin-resident antigen-presenting cells: Instruction manual for vaccine development. Front. Immunol..

[5] Clausen B.E., Kel J.M. (2010). Langerhans cells: Critical regulators of skin immunity?. Immunol. Cell Biol..

[6] Heath W.R., Carbone F.R. (2009). Dendritic cell subsets in primary and secondary T cell responses at body surfaces. Nat. Immunol..

[7] Ueno H., Klechevsky E., Morita R., Aspord C., Cao T., Matsui T., di Pucchio T., Connolly J., Fay J.W., Pascual V. (2007). Dendritic cell subsets in health and disease. Immunol. Rev..

[8] Haniffa M., Gunawan M., Jardine L. (2015). Human skin dendritic cells in health and disease. J. Dermatol. Sci..

[9] Heath W.R., Carbone F.R. (2013). The skin-resident and migratory immune system in steady state and memory: Innate lymphocytes, dendritic cells and T cells. Nat. Immunol..

[10] Elnekave M., Furmanov K., Nudel I., Arizon M., Clausen B.E., Hovav A.H. (2010). Directly transfected langerin^+^ dermal dendritic cells potentiate CD8^+^ T cell responses following intradermal plasmid DNA immunization. J. Immunol..

[11] Lambert P.H., Laurent P.E. (2008). Intradermal vaccine delivery: Will new delivery systems transform vaccine administration?. Vaccine.

[12] Nicolas J.F., Guy B. (2008). Intradermal, epidermal and transcutaneous vaccination: From immunology to clinical practice. Expert Rev. Vaccines.

[13] Picot V. (2008). Intradermal immunization: An alternative route for vaccine administration. Articles as per sessions meeting report. Vaccine.

[14] Vankerckhoven V., van Damme P. (2010). Clinical studies assessing immunogenicity and safety of intradermally administered influenza vaccines. Expert Opin. Drug Deliv..

[15] Bachy V., Hervouet C., Becker P.D., Chorro L., Carlin L.M., Herath S., Papagatsias T., Barbaroux J.B., Oh S.J., Benlahrech A. (2013). Langerin negative dendritic cells promote potent CD8^+^ T-cell priming by skin delivery of live adenovirus vaccine microneedle arrays. Proc. Natl. Acad. Sci. USA.

[16] DeMuth P.C., Min Y., Huang B., Kramer J.A., Miller A.D., Barouch D.H., Hammond P.T., Irvine D.J. (2013). Polymer multilayer tattooing for enhanced DNA vaccination. Nat. Mater..

[17] Chiu Y.N., Sampson J.M., Jiang X., Zolla-Pazner S.B., Kong X.P. (2012). Skin tattooing as a novel approach for DNA vaccine delivery. J. Vis. Exp..

[18] Oosterhuis K., van den Berg J.H., Schumacher T.N., Haanen J.B. (2012). DNA vaccines and intradermal vaccination by DNA tattooing. Curr. Top. Microbiol. Immunol..

[19] Van den Berg J.H., Oosterhuis K., Schumacher T.N., Haanen J.B., Bins A.D. (2014). Intradermal vaccination by DNA tattooing. Methods Mol. Biol..

[20] Wagemakers A., Mason L.M., Oei A., de Wever B., van der Poll T., Bins A.D., Hovius J.W. (2014). Rapid outer-surface protein C DNA tattoo vaccination protects against *Borrelia afzelii* infection. Gene Ther..

[21] Babiarova K., Kutinova L., Zurkova K., Krystofova J., Brabcova E., Hainz P., Musil J., Nemeckova S. (2012). Immunization with WT1-derived peptides by tattooing inhibits the growth of TRAMP-C2 prostate tumor in mice. J. Immunother..

[22] Bins A.D., Jorritsma A., Wolkers M.C., Hung C.F., Wu T.C., Schumacher T.N., Haanen J.B. (2005). A rapid and potent DNA vaccination strategy defined by *in vivo* monitoring of antigen expression. Nat. Med..

[23] Pokorna D., Rubio I., Muller M. (2008). DNA-vaccination via tattooing induces stronger humoral and cellular immune responses than intramuscular delivery supported by molecular adjuvants. Genet. Vaccines Ther..

[24] Verstrepen B.E., Bins A.D., Rollier C.S., Mooij P., Koopman G., Sheppard N.C., Sattentau Q., Wagner R., Wolf H., Schumacher T.N. (2008). Improved HIV-1 specific T-cell responses by short-interval DNA tattooing as compared to intramuscular immunization in non-human primates. Vaccine.

[25] Potthoff A., Schwannecke S., Nabi G., Hoffmann D., Grunwald T., Wildner O., Brockmeyer N.H., Uberla K., Tenbusch M. (2009). Immunogenicity and efficacy of intradermal tattoo immunization with adenoviral vector vaccines. Vaccine.

[26] Daemen T., Riezebos-Brilman A., Regts J., Dontje B., van der Zee A., Wilschut J. (2004). Superior therapeutic efficacy of alphavirus-mediated immunization against human papilloma virus type 16 antigens in a murine tumour model: Effects of the route of immunization. Antivir Ther..

[27] Daemen T., Regts J., Holtrop M., Wilschut J. (2002). Immunization strategy against cervical cancer involving an alphavirus vector expressing high levels of a stable fusion protein of human papillomavirus 16 E6 and E7. Gene Ther..

[28] Van den Berg J.H., Nujien B., Beijnen J.H., Vincent A., van Tinteren H., Kluge J., Woerdeman L.A., Hennink W.E., Storm G., Schumacher T.N. (2009). Optimization of intradermal vaccination by DNA tattooing in human skin. Hum. Gene Ther..

[29] Kis E.E., Winter G., Myschik J. (2012). Devices for intradermal vaccination. Vaccine.

[30] Chen M., Barnfield C., Naslund T.I., Fleeton M.N., Liljestrom P. (2005). MyD88 expression is required for efficient cross-presentation of viral antigens from infected cells. J. Virol..

[31] Huckriede A., Bungener L., Holtrop M., de Vries J., Waarts B.L., Daemen T., Wilschut J. (2004). Induction of cytotoxic T lymphocyte activity by immunization with recombinant Semliki Forest virus: Indications for cross-priming. Vaccine.

[32] Bungener L., Serre K., Bijl L., Leserman L., Wilschut J., Daemen T., Machy P. (2002). Virosome-mediated delivery of protein antigens to dendritic cells. Vaccine.

[33] Schulz O., Diebold S.S., Chen M., Naslund T.I., Nolte M.A., Alexopoulou L., Azuma Y.T., Flavell R.A., Liljestrom P., Reis e Sousa C. (2005). Toll-like receptor 3 promotes cross-priming to virus-infected cells. Nature.

[34] Belz G.T., Smith C.M., Eichner D., Shortman K., Karupiah G., Carbone F.R., Heath W.R. (2004). Cutting edge: Conventional CD8alpha^+^ dendritic cells are generally involved in priming CTL immunity to viruses. J. Immunol..

[35] Allan R.S., Smith C.M., Belz G.T., van Lint A.L., Wakim L.M., Heath W.R., Carbone F.R. (2003). Epidermal viral immunity induced by CD8 alpha^+^ dendritic cells but not by Langerhans cells. Science.

[36] Shortman K., Heath W.R. (2010). The CD8^+^ dendritic cell subset. Immunol. Rev..

[37] Allan R.S., Waithman J., Bedoui S., Jones C.M., Villadangos J.A., Zhan Y., Lew A.M., Shortman K., Heath W.R., Carbone F.R. (2006). Migratory dendritic cells transfer antigen to a lymph node-resident dendritic cell population for efficient CTL priming. Immunity.

[38] Stoitzner P., Tripp C.H., Eberhart A., Price K.M., Jung J.Y., Bursch L., Ronchese F., Romani N. (2006). Langerhans cells cross-present antigen derived from skin. Proc. Natl. Acad. Sci. USA.

[39] Bachmann M.F., Jennings G.T. (2010). Vaccine delivery: A matter of size, geometry, kinetics and molecular patterns. Nat. Rev. Immunol..

[40] Cubas R., Zhang S., Kwon S., Sevick-Muraca E.M., Li M., Chen C., Yao Q. (2009). Virus-like particle (VLP) lymphatic trafficking and immune response generation after immunization by different routes. J. Immunother..

[41] Johnston L.J., Halliday G.M., King N.J. (2000). Langerhans cells migrate to local lymph nodes following cutaneous infection with an arbovirus. J. Investig. Dermatol..

[42] Froelich S., Tai A., Kennedy K., Zubair A., Wang P. (2011). Virus-receptor mediated transduction of dendritic cells by lentiviruses enveloped with glycoproteins derived from Semliki Forest virus. PLOS ONE.

[43] De Witte L., Nabatov A., Geijtenbeek T.B. (2008). Distinct roles for DC-SIGN^+^-dendritic cells and Langerhans cells in HIV-1 transmission. Trends Mol. Med..

[44] Kissenpfennig A., Henri S., Dubois B., Laplace-Builhe C., Perrin P., Romani N., Tripp C.H., Douillard P., Leserman L., Kaiserlian D. (2005). Dynamics and function of Langerhans cells *in vivo*: Dermal dendritic cells colonize lymph node areas distinct from slower migrating Langerhans cells. Immunity.

[45] Van den Berg J.H., Quaak S.G., Beijnen J.H., Hennink W.E., Storm G., Schumacher T.N., Haanen J.B., Nuijen B. (2010). Lipopolysaccharide contamination in intradermal DNA vaccination: Toxic impurity or adjuvant?. Int. J. Pharm..

[46] Harden L.M., du Plessis I., Poole S., Laburn H.P. (2006). Interleukin-6 and leptin mediate lipopolysaccharide-induced fever and sickness behavior. Physiol. Behav..

[47] Yoshizumi M., Nakamura T., Kato M., Ishioka T., Kozawa K., Wakamatsu K., Kimura H. (2008). Release of cytokines/chemokines and cell death in UVB-irradiated human keratinocytes, HaCaT. Cell Biol. Int..

[48] Gopee N.V., Cui Y., Olson G., Warbritton A.R., Miller B.J., Couch L.H., Wamer W.G., Howard P.C. (2005). Response of mouse skin to tattooing: Use of SKH-1 mice as a surrogate model for human tattooing. Toxicol. Appl. Pharmacol..

[49] Byrne S.N., Halliday G.M., Johnston L.J., King N.J. (2001). Interleukin-1beta but not tumor necrosis factor is involved in West Nile virus-induced Langerhans cell migration from the skin in C57BL/6 mice. J. Invest. Dermatol..

[50] Oosterhoff D., Heusinkveld M., Lougheed S.M., Kosten I., Lindstedt M., Bruijns S.C., van Es T., van Kooyk Y., van der Burg S.H., de Gruijl T.D. (2013). Intradermal delivery of TLR agonists in a human explant skin model: Preferential activation of migratory dendritic cells by polyribosinic-polyribocytidylic acid and peptidoglycans. J. Immunol..

[51] Schneider L.P., Schoonderwoerd A.J., Moutaftsi M., Howard R.F., Reed S.G., de Jong E.C., Teunissen M.B. (2012). Intradermally administered TLR4 agonist GLA-SE enhances the capacity of human skin DCs to activate T cells and promotes emigration of Langerhans cells. Vaccine.

[52] Fehres C.M., Bruijns S.C., van Beelen A.J., Kalay H., Ambrosini M., Hooijberg E., Unger W.W., de Gruijl T.D., van Kooyk Y. (2014). Topical rather than intradermal application of the TLR7 ligand imiquimod leads to human dermal dendritic cell maturation and CD8^+^ T-cell cross-priming. Eur. J. Immunol..

[53] Polak M.E., Newell L., Taraban V.Y., Pickard C., Healy E., Friedmann P.S., al-Shamkhani A., Ardern-Jones M.R. (2012). CD70-CD27 interaction augments CD8^+^ T-cell activation by human epidermal Langerhans cells. J. Investig. Dermatol..

[54] Flacher V., Tripp C.H., Mairhofer D.G., Steinman R.M., Stoitzner P., Idoyaga J., Romani N. (2014). Murine Langerin^+^ dermal dendritic cells prime CD8^+^ T cells while Langerhans cells induce cross-tolerance. EMBO Mol. Med..

